# Clinically proven natural products against breast cancer, with mechanistic insights

**DOI:** 10.32604/or.2025.062778

**Published:** 2025-06-26

**Authors:** MD. MAHMUDUL HASAN, SHAH MD. WASIN, MISHU RAHMAN, EVA AZME, MD. SAQLINE MOSTAQ, MD. MAHEDI HASAN NAHID, NOR MOHAMMAD, FARJANA AFRIN TANJUM, MD. ANAMUL HAQUE, MD ASHIQ MAHMUD, MOHAMMAD NURUL AMIN

**Affiliations:** 1 Department of Pharmacy, University of Chittagong, Chittagong, 4331, Bangladesh; 2 Department of Natural Products and Pharmacology, Pratyasha Health Biomedical Research Center, Dhaka, 1230, Bangladesh; 3 Department of Pharmacy, Mawlana Bhashani Science and Technology University, Tangail, 1902, Bangladesh; 4 Department of Biochemistry and Molecular Biology, Mawlana Bhashani Science and Technology University, Tangail, 1902, Bangladesh; 5 School of Basic Pharmaceutical and Toxicological Sciences, College of Pharmacy, University of Louisiana at Monroe, Monroe, LA 71201, USA; 6 Department of Pharmacy, University of Asia Pacific, Dhaka, 1205, Bangladesh; 7 Department of Chemistry, University of Chittagong, Chattogram, 4331, Bangladesh; 8 Bachelor of Medicine and Bachelor of Surgery, Chattogram Maa-o-Shishu Hospital Medical College, Agrabad, Chattogram, 4100, Bangladesh

**Keywords:** Breast cancer, Natural products, Chemotherapeutic potential

## Abstract

**Background:**

Breast cancer still stands to be the foremost contributor to cancer-related incidence and mortality in women globally accounting for about 14% of all female cancer-related deaths worldwide. This research seeks to illustrate the mechanisms and clinical findings of natural products against breast cancer treatment.

**Methodology:**

Required data for this review article was retrieved employing several readily obtainable search databases, including Web of Science^®^ (Thomson Reuters, USA), PubMed^®^ (U.S. National Library of Medicine, USA), and SciVerse Scopus^®^ (Elsevier Properties S.A., USA), taking into consideration certain search terms like “breast cancer,” “natural products against breast cancer,” and “Clinically proven natural products in the treatment of breast cancer” and so on.

**Results:**

Several natural products, namely Omega-3 fatty acids, dietary isothiocyanates, curcumin, green tea, flaxseed, limonene, and others, were found to modulate crucial pathways in breast cancer cells. These substances suppressed angiogenesis by downregulating the vascular endothelial growth factor (VEGF), promoted apoptosis by activating caspase enzymes, and prevented cell proliferation by controlling cyclin-dependent kinases. Clinical studies demonstrated improved outcomes in patients receiving these natural products with standard treatment procedures.

**Discussion:**

The findings underscore the multifaceted functions of natural products in breast cancer therapy, highlighting their potential to increase the efficacy of conventional treatments while reducing adverse effects. Further exploration of synergistic actions and optimal dosages is needed.

**Conclusion:**

Clinically proven natural products represent a potential avenue for breast cancer treatment with their mechanistic insights that facilitate their incorporation into treatment regimens. To maximize clinical applications, future inquiries should center on elucidating the full spectrum of these anticancer functions.

## Introduction

Breast cancer, a heterogeneous group of malignancies initiated from breast tissue, is usually characterized by the existence of a palpable lump or mass. Predominantly, this malignancy originates from epithelial cells that line the milk ducts [1]. The World Health Organization highlights that breast cancer imposes a considerable global health risk, with above 2.3 million emerging cases recorded annually and over 670 thousand deaths attributed to the disease in 2022 alone. This fatality rate accounts for about 14% of all female cancer-related fatalities worldwide [2].

The progression of breast cancer relies on the intricate synchronization of several mechanisms within cancer cells and their surrounding milieu. The sequential cascade of genetic alterations, which disrupts the control of cell division and apoptosis, is universally agreed as the primary instigating event in the initiation of cancer [1]. Initiation comprises genetic changes affecting genes such as breast cancer gene-1 (BRCA1) [2], breast cancer gene-2 (BRCA2), and tumor protein p53 (TP53), which can be either inherited or acquired [3]. Hormonal effects, particularly estrogen and progesterone, encourage Cellular proliferation, notably in estrogen receptor-positive (ER+) breast cancer [4]. As the disease advances, genetic mutations mount up, leading to the progression of a localized tumor with invasive potentiality [5]. As a result of intricate synergy between cancer cells and their microenvironment, metastasis develops as well as allows for the spread of the disease to other locations [6]. In triple-negative breast cancer (TNBC), characterized by the absence of estrogen receptor (ER), progesterone receptor (PR), and human epidermal growth factor receptor 2 (HER2) expression, cell proliferation may be fueled by alternative pathways namely PI3K/AKT/mTOR [7]. On top of that, the development of breast cancer can be influenced by epigenetic changes, angiogenesis, dysregulation of the immune system, tumor interactions with the surroundings milieu, and dysregulation of cellular senescence and apoptosis [8–12].

Treatment approaches for breast cancer differ according to molecular subgroups, highlighting the heterogeneity of the cancer [13]. Multidisciplinary techniques are used in management to combine systemic treatments that target the entire body with locoregional therapies like radiation and surgery [4,14]. These consist of anti-HER2 therapy for HER2-positive disease, hormone therapy for hormone receptor-positive disease, chemotherapy, and developing immunotherapy [15–17]. While HER2-positive breast cancer benefits from medications such as trastuzumab, hormone-positive breast cancer responds to hormone treatment that targets estrogen or progesterone receptors [18,19]. However, the inadequacy of hormone receptors and HER2 expression in TNBC, which accounts for 15%–20% of cases, presents difficulties and limits available therapy choices [15]. The aggressive nature of TNBC prompts extensive investigation into therapeutic options, and immune checkpoint inhibitors have demonstrated potential in immunotherapy [13]. Apart from chemotherapy and radiation therapy, these medical interventions may result in significant adverse reactions and patients may acquire resistance. While the natural products showed significant outcomes against breast cancer without significant adverse effects. Moreover, there are no established therapies for TNBC. Thus, it is essential to have a thorough grasp of creating novel, targeted, and efficient treatments for every subgroup of breast cancer [20].

Natural products have displayed notable promise in the battle against breast cancer, owing to their effectiveness in targeting particular mechanisms linked to carcinogenesis. Polyphenols, occurring abundantly in fruits and vegetables, disclose anti-breast cancer effects by modifying important signaling pathways [21]. Renowned phytochemicals, flavonoids, and lignans intervene in the expansion of tumors by regulating the cell cycle, initiating apoptosis, and inhibiting angiogenesis [22]. Mechanistically, flavonoids exert antioxidant activity and block cell proliferation, while lignans intervene with estrogen receptor signaling and retain antiangiogenic effects [23,24]. Furthermore, terpenoids, alkaloids, and saponins derived from medicinal plants exhibit cytotoxic properties against breast cancer cells through their targeting of pathways such as NF-κB, MAPK, and PI3K/Akt. These compounds induce disruption of the cell cycle and initiation of apoptosis inhibiting proliferation and metastasis. There is potential for novel treatments for breast cancer by comprehending the complex interactions between natural products and molecular pathways [25–27].

Therefore, drugs derived from natural sources can be used as drug leads that can be optimized using synthetic means. Only clinically proven natural products are suitable candidates for developing new drugs and can make significant contributions to drug development and drug design. In this review, the authors had a specific aim to explain only clinically established natural products in the management of breast cancer, providing mechanistic insights.

## Methodology

**Figure 1:**
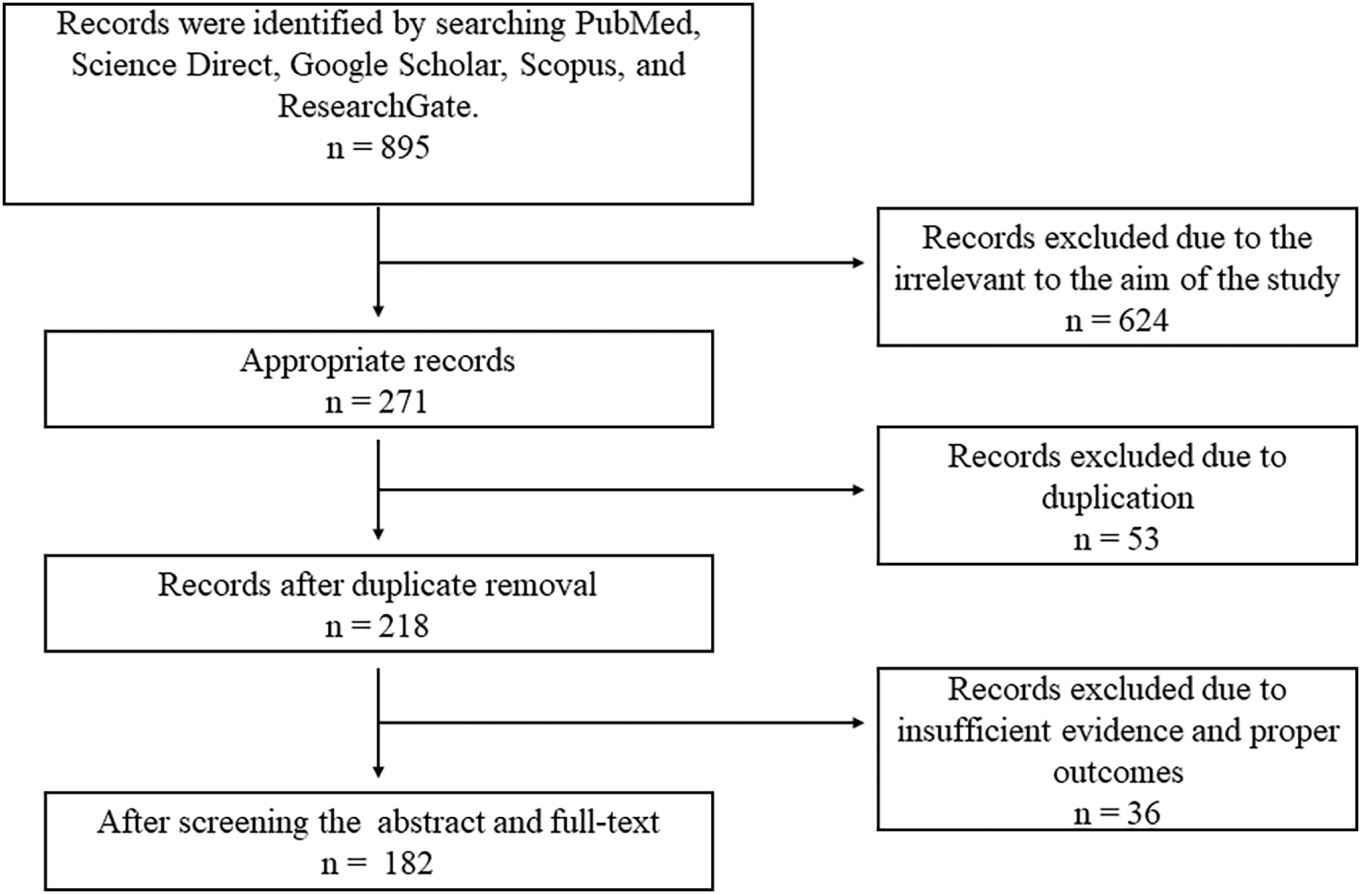
Diagram illustrating the workflow of literature retrieval and selection.

For the present comprehensive review, a specific keyword-driven search was performed to retrieve information from the following online databases including Web of Science^®^ (Thomson Reuters, USA), PubMed^®^ (U.S. National Library of Medicine, USA), and SciVerse Scopus^®^ (Elsevier Properties S.A., USA). Common search terms include “breast cancer,” “natural products against breast cancer,” and “clinically proven natural products in the treatment of breast cancer”. A total of 895 papers were found. After a preliminary screening, 218 of the 271 non-duplicate items from the initial search were determined to be relevant. A sum of 182 papers were chosen for a thorough review after 36 were excluded. Only clinical and scientific studies written in English were included. Articles lacking clinical evidence were excluded. Each article’s title, abstract, and conclusions were thoroughly examined to make sure they met the study’s inclusion requirements and were relevant (Fig. 1). From the final list of articles, natural products found effective against breast cancer based on their clinical outcomes were discussed in this study and summarized in Table 1.

**Table 1:** Summary of clinically proven natural products against breast cancer

Natural product name	Title of the source	Number of patients	Mechanism of action	Summary of findings	Toxicity	Ref.
Omega-3 fatty acids	Erythrocyte fatty acids and risk of proliferative and nonproliferative fibrocystic disease in women in Shanghai, China	155 women suffering from non-proliferative fibrocystic changes 185 women with proliferative fibrocystic changes & 241 with breast cancer, 1030 controls.	Induction apoptosis via Bcl2/Bax, disruption Wnt/β-catenin, G1 arrest, suppression growth.	Significantly decreased 67% of non-proliferative fibrocystic changes risk; inverse association with benign and proliferative changes.	No significant toxicity.	[34]
Dietary Isothiocyanates	A Presurgical-Window Intervention Trial of Isothiocyanate-Rich Broccoli Sprout Extract in Patients with Breast Cancer	30 patients of postmenopausal breast cancer in clinical trial.	G2-M arrest, oxidative DNA damage, ATM/ATR activation, inhibition EGFR/HER2.	Notable anticancer effects with exceptional compliance.	Negligible	[46]
Onion and Garlic	Garlic, onion and cereal fiber as protective factors for breast cancer: A French case-control study	345 breast cancer cases (northeast France).	Induction apoptosis, inhibition of histone deacetylases (garlic); blockage of Cdks, and Fas-mediated apoptosis (onions).	Significant risk reduction (*p* < 10^-6) among garlic/onion consumers.	No toxicity	[54]
Isoflavones	Soy isoflavones decrease fibro glandular breast tissue measured by magnetic resonance imaging in premenopausal women: A 2-year randomized double-blind placebo-controlled clinical trial	197 premenopausal breast cancer individuals in a randomized trial.	Targeting ER, PI3K/Akt/mTOR, decreasing mTOR activation, alteration IGF-1R signaling.	Reduction of FGBT, a breast cancer biomarker.	Generally well-tolerated	[64]
*Viscum album* L.	Efficacy and Safety of Long-term Complementary Treatment with Standardised European Mistletoe Extract (*Viscum album* L.) in addition to the Conventional Adjuvant Oncological Therapy in Patients with Primary Non-metastatic Breast Cancer	1442 breast cancer patients (710 test, 732 control).	Induction of apoptosis, releasing immune cytokines, inhibition of protein synthesis (ML-I).	Improved symptoms, and longer survival (HR = 0.46, *p* = 0.038).	Mild injection site reactions were reported.	[74]
*Salvia miltiorrhiza*	Danshen Improves Survival of Patients with Breast Cancer and Dihydroisotanshinone I Induces Ferroptosis and Apoptosis of Breast Cancer Cells	79,335 metastatic breast cancer patients in Taiwan.	Inhibition PI3K/Akt, induction of apoptosis, G1/M arrest via p21/p27.	Lower mortality rates (*p* < 0.001) with >84 g use after diagnosis.	No significant toxicity was reported.	[81]
Curcumin	Efficacy and safety of curcumin in combination with paclitaxel in patients with advanced, metastatic breast cancer: A comparative, randomized, double-blind, placebo-controlled clinical trial	150 women suffering from advanced & metastatic breast cancer.	Suppression of tumor growth, angiogenesis, induction apoptosis, regulation Akt/mTOR.	Higher ORR (51% vs. 33%, *p* < 0.01) after four weeks.	Well-tolerated.	[88]
Green Tea	Effects of a green tea extract, Polyphenon E, on systemic biomarkers of growth factor signaling in women with hormone receptor-negative breast cancer	40 women with breast cancer in Phase IB trial post-adjuvant treatment.	Inhibition MAPK/AKT pathways, induction G1/M and G2/M arrest, activation pro-apoptotic proteins.	Potential to suppress growth factor signaling, angiogenesis, and lipid metabolism.	Mild GI discomfort in some patients.	[110]
*Scutellaria barbata*	Molecular mechanisms underlying selective cytotoxic activity of BZL101, an extract of Scutellaria barbata, towards breast cancer cells	21 advanced breast cancer patients in Phase I trial.	Cytotoxic via oxidative DNA damage, PARP activation, ROS increase, and ATP depletion.	No severe side effects; further research is needed for metastatic breast cancer therapy.	No grade III/IV toxicities were reported.	[119]
*Trametes versicolor*	Phase 1 Clinical Trial of *Trametes versicolor* in Women with Breast Cancer	11 breast cancer patients’ post-radiation therapy in Phase I trial.	Boosting pro-apoptotic proteins, enhancing immune response, inducing apoptosis.	Safe with the potential to restore immune function.	Well-tolerated.	[123]
Limonene	Human Breast Tissue Disposition and Bioactivity of Limonene in Women with Early-Stage Breast Cancer	43 early-stage breast cancer patients.	Induction apoptosis via Bax activation, blocking VEGF, inhibition Akt/Myc & G1 arrest.	Cyclin D1 expression reduced by 22% (*p* = 0.002).	No significant toxicity was reported.	[130]
Flaxseed	Dietary Flaxseed Alters Tumor Biological Markers in Postmenopausal Breast Cancer	32 postmenopausal breast cancer patients in the placebo-controlled trial.	Production of enterodiol, binding to estrogen receptors & blocking estrogen action in ER+ cells.	Reduced Ki-67 (34.2%; *p* = 0.001), increased apoptosis (30.7%; *p* = 0.007).	No toxicity was reported.	[138]

## Natural Products Widely Utilized in the Management of Breast Cancer

### Omega-3 fatty acids

Omega-3 fatty acids, vital polyunsaturated fats, are crucial nutrients obtained solely from the diet, primarily occurring in fatty fish like salmon and tuna, along with fish oil supplements [28]. It serves an essential function in altering blood lipid profiles, influencing various health issues including cardiovascular disease, diabetes, and cancer, along with mental illnesses, and aiding brain and eye function in pregnant and lactating women and infants [29,30]. Additionally, it exhibits therapeutic efficacy against a variety of cancers, such as breast cancer, colorectal cancer, leukemia, gastric cancer, pancreatic cancer, esophageal cancer, prostate cancer, lung cancer, head and neck cancer, and cancer cachexia [31,32]. High ratios of omega-3 fatty acids eicosapentaenoic acid (EPA) and docosahexaenoic acid (DHA) may decrease breast cancer risk through inhibiting inflammation and growth factor signaling; simultaneously, supplementing EPA and DHA is investigated for addressing post-breast cancer issues [33].

#### Clinical study

A case-control study included 155 women suffering from non-proliferative fibrocystic breast changes (NPFCs), 185 with proliferative changes (PFCs), 241 suffering from breast cancer (127 with non-proliferative and 114 with proliferative alterations in extra tumoral mammary gland epithelium), and 1030 controls. It aimed to analyze erythrocyte fatty acid concentrations. The comparative risk for NPFCs, and PFCs, in conjunction with breast cancer both proliferative and non-proliferative alterations in extra tumoral tissue, was assessed. Higher eicosapentaenoic acid (EPA) levels correlated with 67% lower NPFC risk; Omega-3 (n–3) fatty acids intake and Palmitic: palmitoleic acid (n–7 saturation index) were negatively correlated with benign and proliferative breast changes, potentially mitigating breast cancer progression [34].

#### Mechanistic view

**Figure 2:**
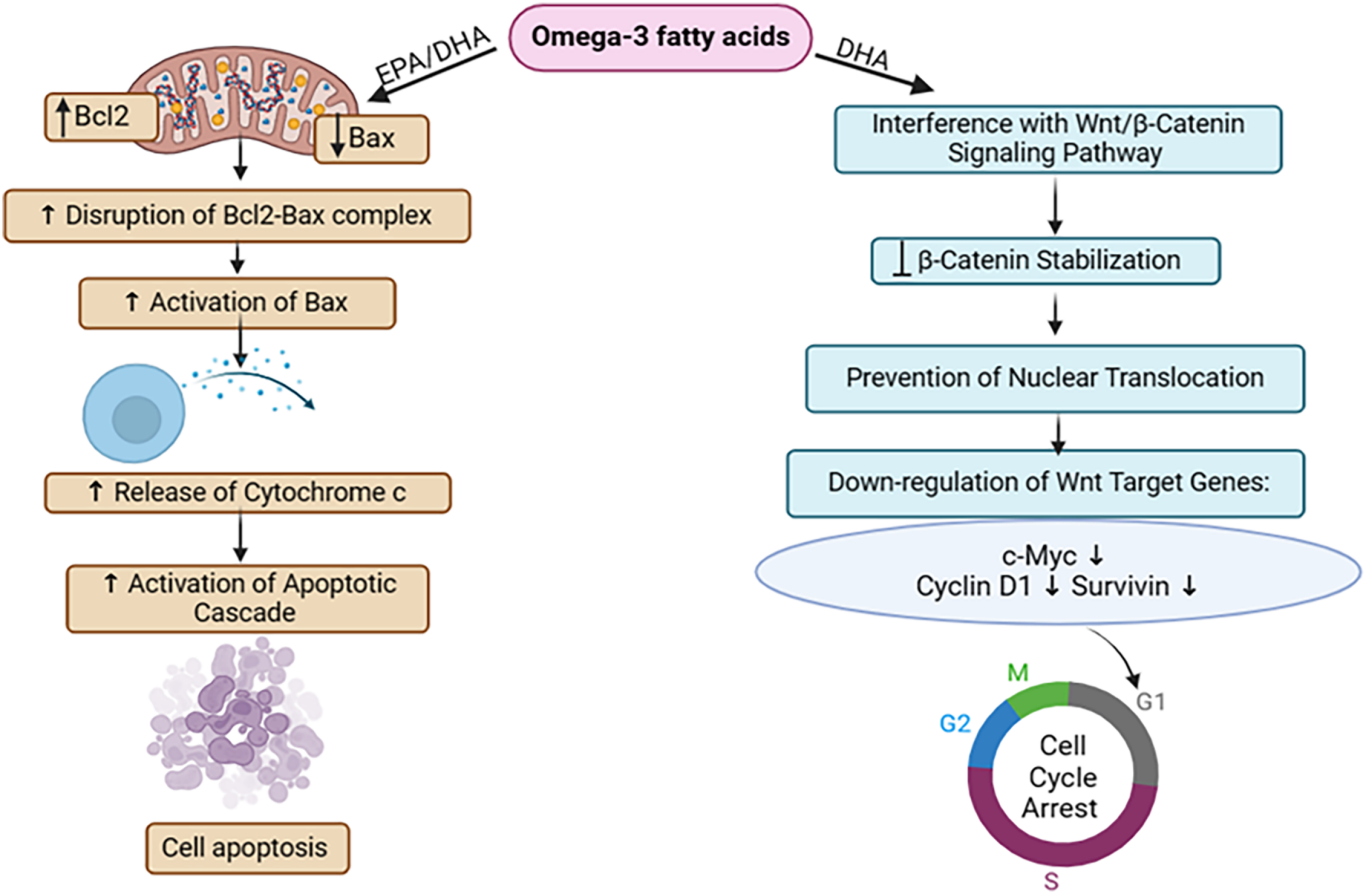
The mode of action of omega-3 fatty acids against breast Cancer: Through the regulation of Bcl2 and Bax proteins and disruption of the Wnt/β-catenin pathway, omega-3 fatty acids initiate apoptosis in breast cancer cells. It also decreases cellular proliferation via G1 cell cycle arrest, thus suppressing tumor growth in breast cancer [EPA = Eicosapentaenoic Acid, DHA = Docosahexaenoic Acid, Bcl2 = B-cell Lymphoma 2, Bax = Bcl-2-associated X protein, Wnt = Wingless-related Integration Site, β-Catenin = Beta-Catenin, c-Myc = Cellular Myelocytomatosis Oncogene]. Credit: bioRender.

EPA/DHA, which are omega-3 fatty acids, influence the extent of expression of two key proteins implicated in apoptosis regulation: Bcl2 and Bax. By modulating their expression, EPA/DHA alters the equilibrium between pro-survival (Bcl2) and pro-apoptotic (Bax) signals, favoring apoptosis. This alteration disrupts the interaction between Bcl2 and Bax proteins, allowing Bax to become activated. Activated Bax then stimulates the excretion of cytochrome c from mitochondria, a pivotal event triggering the apoptotic cascade. Ultimately, this cascade Initiates apoptosis in the breast in MDA-MB-231 breast cancer cells [35,36]. Also, DHA, a component of omega-3 fatty acids, suppresses breast cancer cell multiplication by targeting the Wnt/β-catenin signaling pathway. Normally, this pathway promotes cell proliferation and survival by activating specific genes. However, DHA, an omega-3 fatty acid, disrupts β-catenin stabilization, reducing its translocation into the nucleus. This interference affects the initiation of genes associated with cell functions like cell growth and survival. The described process leads to decreased activation of key genes engaged in cell cycle advancement (such as c-Myc and cyclin D1) and anti-apoptotic pathways (such as survivin). Consequently, DHA induces cell cycle arrest, primarily at the G1 phase, and promotes apoptosis. These effects hinder breast cancer cell expansion within laboratory settings as well as animal models, ultimately slowing tumor progression and potentially halting breast cancer advancement [37] (Fig. 2).

### Dietary isothiocyanates (cruciferous vegetable)

Isothiocyanates (ITCs) are bioactive chemicals generated from plants through the enzymatic breakdown of glucosinolates (GLs), mainly present in cruciferous vegetables. These stress-response compounds, which are plentiful in plants of the Cruciferae family, are some of the predominant secondary bioactive compounds in the Brassicales botanical order [38–40]. ITCs showcase diverse biological functions including anticancer, anti-inflammatory, and antioxidant effects. Additionally, they display antibacterial, antifungal, and antiviral properties while mitigating oxidative stress as indirect antioxidants. Moreover, ITCs contribute to an array of health-promoting properties including anticancerous, neuroprotective, and cardio-protective effects in humans [41,42]. Dietary isothiocyanate has demonstrated efficacy in treating various cancers affecting the lung, esophagus, stomach, colon, breast, bladder, pancreas, and prostate [39,43,44]. Additionally, there’s a notable connection between ITC concentrations and a minimized risk of breast cancer affecting pre- and postmenopausal women. Moreover, ITC has demonstrated a noteworthy suppressive effect on breast cancer stem cells, introducing an unexplored avenue to the treatment of chemo-resistant breast cancer [45].

#### Clinical study

Thirty postmenopausal breast cancer individuals were recruited in a clinical study, in which they were arbitrarily designed to receive either 200 µmol of isothiocyanate (ITC) daily from broccoli sprout extract (BSE) or a placebo over the course of two weeks before undergoing surgery. Immunohistochemistry staining was employed to assess biomarker expressions associated with chemo-resistant breast cancer ITC function in breast cancer tissue specimens. The trial showcased exceptional compliance, with all participants adhering to their assigned regimen, and negligible toxicity, with no occurrences of grade 4 adverse events. Remarkably, the BSE group demonstrated notable anticancer effects [46].

#### Mechanistic view

**Figure 3:**
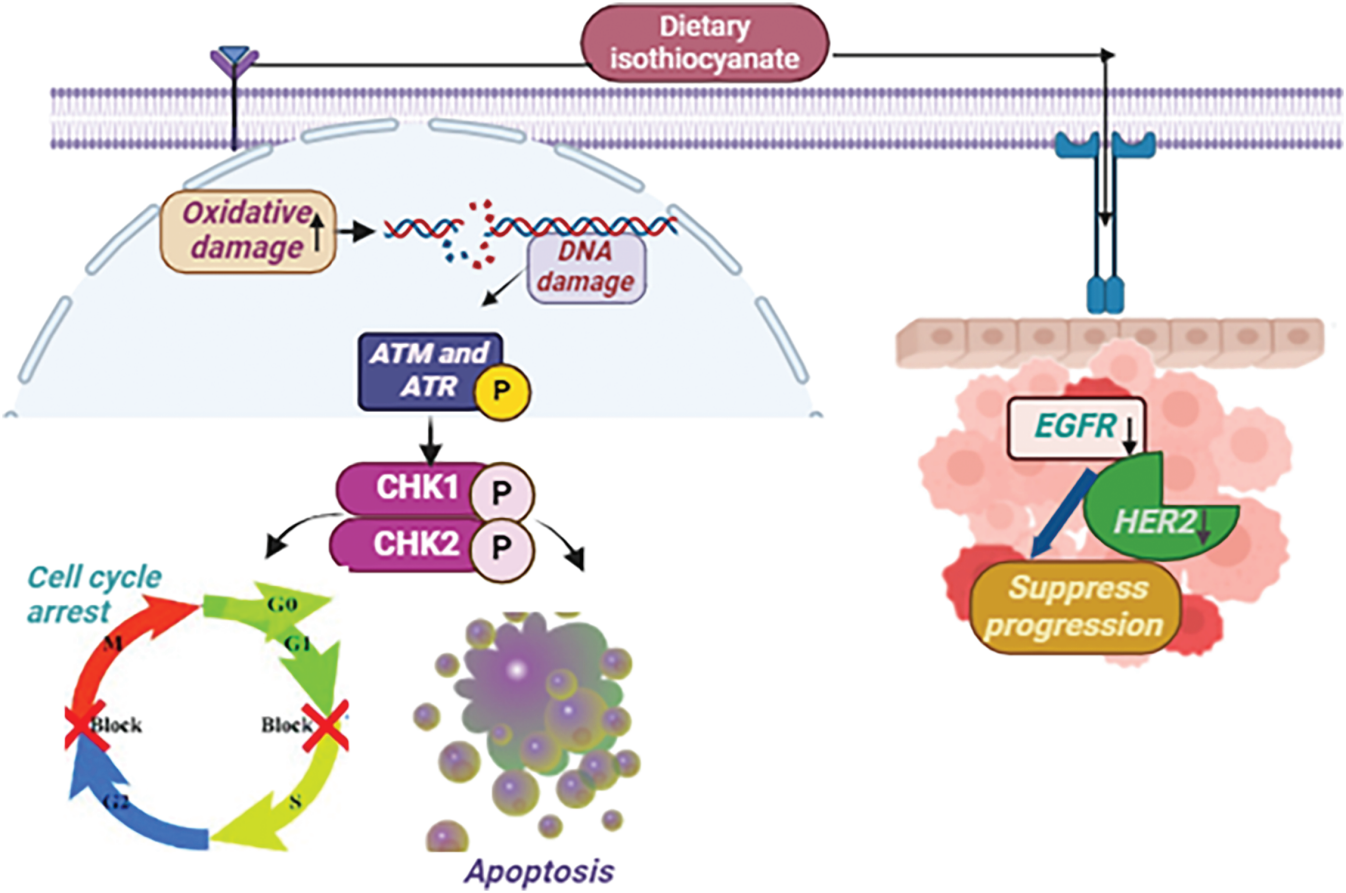
The mode of action of Dietary isothiocyanates against breast Cancer: Sulforaphane, an isothiocyanate present in cruciferous vegetables, suppresses breast cancer growth by causing a G2-M cell cycle halt and increasing oxidative DNA damage. It stimulates ATM and ATR kinases, causing CHK1/CHK2 phosphorylation, which promotes cell cycle arrest or apoptosis. Sulforaphane also suppresses EGFR along with HER2 receptor activation, hence slowing breast cancer growth [EGF= Epidermal Growth Factor Receptor, HER2 = Human Epidermal Growth Factor Receptor-2, CHK1 = Checkpoint Kinase 1, CHK2 = Checkpoint Kinase 2, ATM = ataxia telangiectasia mutated, ATR = ATM and Rad3-related (↑ = increase, ↓ = decrease)]. Credit: bioRender.

Sulforaphane (isothiocyanate), obtained from cruciferous vegetables, has promising capability in fighting breast cancer by inhibiting cancer cell expansion through a G2-M cell cycle arrest in MCF-7 cells [47]. Its electrophilic nature enhances oxidative damage by interacting with DNA, towards the induction of ATM (ataxia telangiectasia mutated) and ATR (ATM and Rad3-) kinases, which are crucial in the DNA damage response. ATM typically detects double-strand lesions, whereas ATR responds to single-stranded DNA regions and replication stress. ATM and ATR become active and phosphorylate CHK1 and CHK2, which then trigger signaling pathways that result in either cell cycle arrest or apoptosis, thereby impeding the advancement of breast cancer [48]. In breast cancer, epidermal growth factor receptors (EGFR) and HER2 receptors serve crucial functions in cell multiplication, viability, along migration. Sulforaphane acts on these receptors by reducing their activity when they interact with a ligand like EGF [49]. This action inhibits subsequent signaling pathways and reduces cell proliferation, showing promise in fighting breast cancer (Fig. 3).

### Garlic and onion

Garlic and onion, part of the *Alliaceae* family under the Genus *Allium* within the larger *Liliaceae* family, consisting of more than 600 species, and are perennial crops that are widely known as essential food items worldwide [50]. Garlic (*Allium sativum*), as well as onion (*Allium cepa*), are widely recognized for their adaptable use in cooking and possess exceptional characteristics. The extracts from both sources possess strong antioxidant, hypocholesterolemia, hypolipidemic, anti-hypertensive, and neuroprotective properties [51–53]. A high diet of garlic and onions acts as a barrier to breast cancer progression [54].

#### Clinical study

An investigation carried out in northeast France spanning from 1986 to 1989, involving 345 individuals diagnosed with breast cancer, unveiled a noteworthy finding: those who ingested garlic or onion experienced a significant risk reduction (*p* < 10^−6^). In light of this discovery, clinical trials Exploring the effectiveness of garlic and onion in treating breast cancer were initiated. The trial focused on evaluating the treatment’s impact on the malignancies and the patients’ overall prognoses onion and [54,55].

#### Mechanistic view of garlic against breast cancer

**Figure 4:**
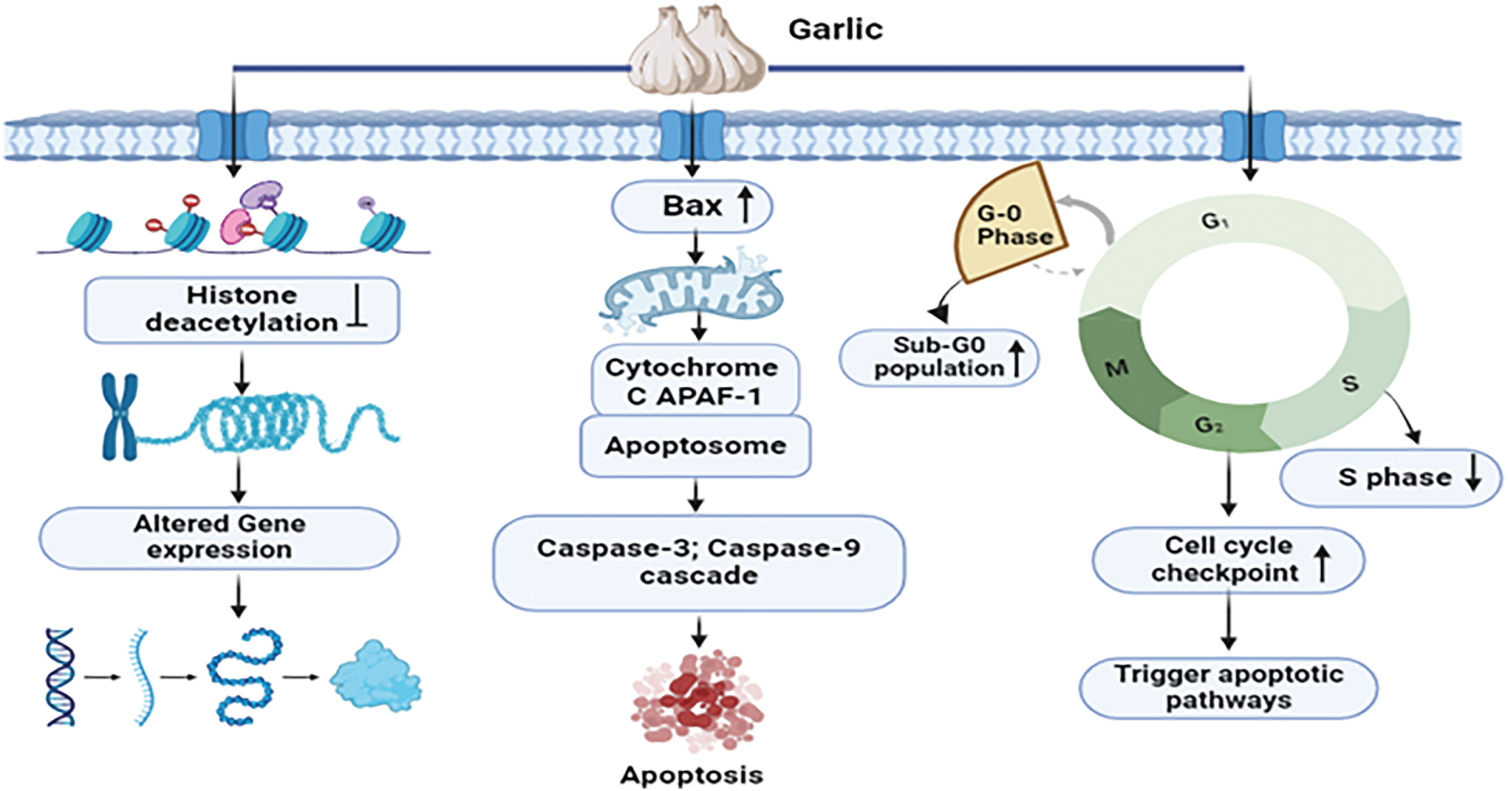
Mechanistic view of garlic against breast cancer: Garlic extract fights against breast cancer by inducing apoptosis via DNA fragmentation and lowering S phase occupancy, which inhibits DNA synthesis. It triggers cell cycle checkpoints and apoptotic pathways, comprising the Bax, cytochrome c, and caspase cascades, resulting in cell death. Furthermore, garlic extract alters the epigenetic landscape by suppressing histone deacetylases, which increases anti-cancer gene expression [Bax = Bcl-2-associated X protein, APAF-1 = Apoptotic Protease Activating Factor 1, G0 Phase = Resting Phase in the Cell Cycle, G1 Phase = Growth Phase 1, S Phase = DNA Synthesis Phase, G2 Phase = Growth Phase 2, M Phase = Mitosis Phase, Caspase-3 = Cysteine-aspartic Acid Protease 3, Caspase-9 = Cysteine-aspartic Acid Protease 9]. Credit: bioRender.

Garlic extract employs a comprehensive mechanistic approach to combat breast cancer. It induces a dose-dependent elevation in the sub-G0 apoptotic population, characterized by DNA fragmentation, a distinctive feature of apoptosis upon application. Simultaneously, the extract impedes the advancement of breast cancer cells by reducing S phase occupancy, thereby inhibiting DNA synthesis in the MCF-7 breast cancer cell. By the activation of cell cycle checkpoints and apoptotic pathways, this interference results in the elimination of abnormal cells. The impact of garlic extract extends to the epigenetic landscape, where it regulates the cell cycle in addition to enhancing the regulation of anti-cancer gene expression by inhibiting histone deacetylases [56]. Furthermore, the extract prompts the release of proapoptotic factors, including Bax and cytochrome c, initiating the formation of the apoptosome and initiating cascades involving caspase-3 and caspase-9. The culmination of these processes involves the DNA double helix and programmed cell death [57]. In summary, garlic extract’s mechanisms against breast cancer are intricate, involving both cellular and epigenetic factors (Fig. 4).

#### Mechanistic view of onion against breast cancer

**Figure 5:**
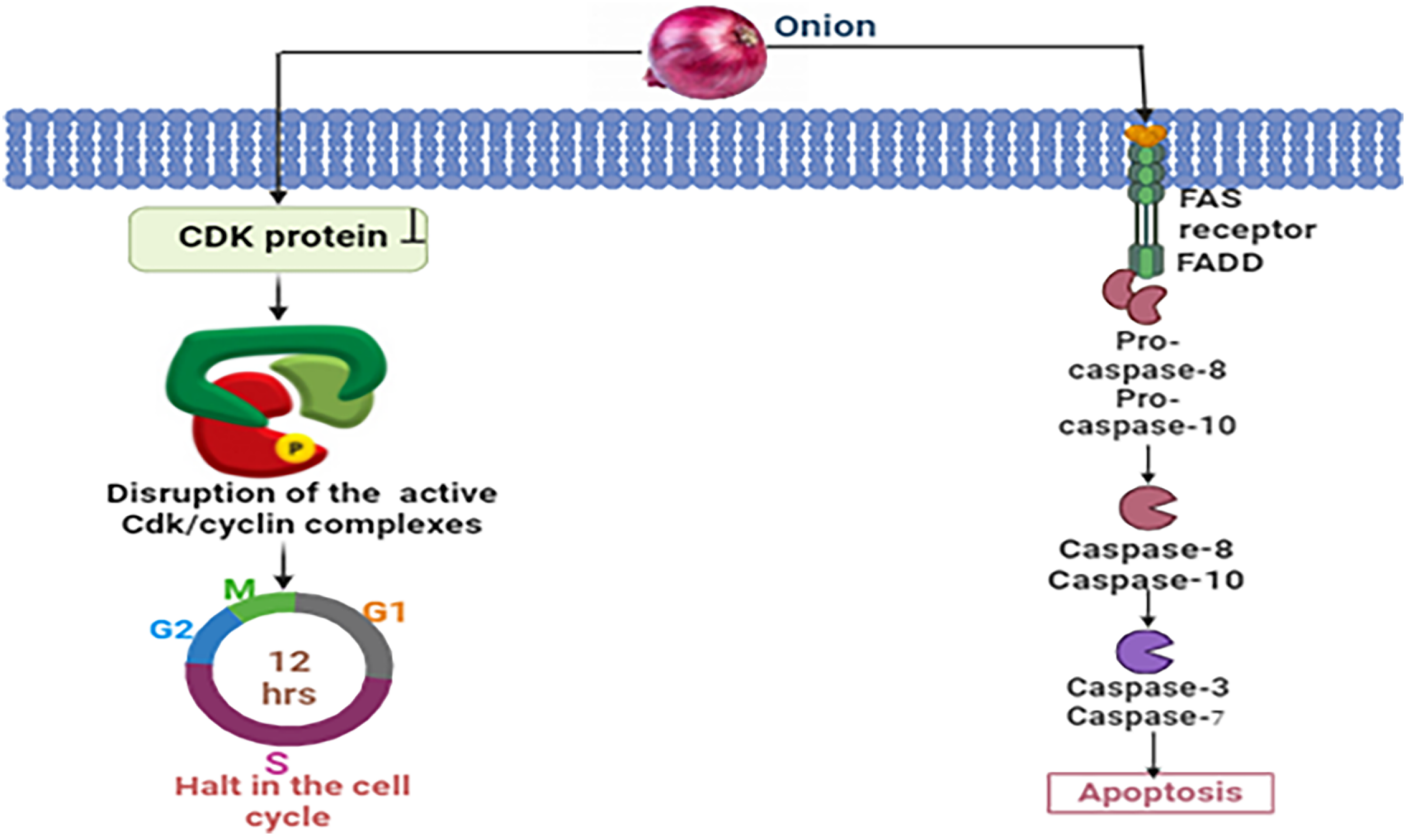
Mechanistic view of onion against breast cancer: Through the binding of Cdks, blockage of their activation and phosphorylation, and disruption of Cdk/cyclin complexes, chemicals derived from onions prevent the progression of breast cancer by halting the cell cycle progression in the G1, S, or G2 phases. Onions also cause apoptosis through activating death receptors involving Fas, which sets off the apoptotic cascade and causes DISC formation and caspase-8 activation [CDK = Cyclin-dependent Kinase, FAS = FAS receptor (also known as CD95 or APO-1), FADD = FAS-associated Death Domain protein, Pro-caspase-8 = Pro-cysteine-aspartic acid protease 8, Pro-caspase-10 = Pro-cysteine-aspartic acid protease 10, Caspase-8 = Cysteine-aspartic acid protease 8, Caspase-10 = Cysteine-aspartic acid protease 10, Caspase-3 = Cysteine-aspartic acid protease 3, Caspase-7 = Cysteine-aspartic acid protease 7]. Credit: bioRender.

By directly binding Cdks and blocking their activation and phosphorylation, organosulfur compounds derived from onions slow the advancement of breast cancer. The interruption of Cdk/cyclin complexes specifically stops the progression of the cell cycle during the G1, S, or G2 phases [58]. In addition, these compounds induce apoptosis by activating death receptors such as Fas, which initiates the production of a death-inducing signaling complex (DISC). Caspase-8 activation, followed by ensuing activation of downstream caspases is essential for regulating programmed cell death mechanisms that combat breast cancer [59] (Fig. 5).

### Isoflavones

Isoflavones are a kind of phytoestrogen that occurs naturally in soybeans along with soy products. These substances are obtained from plants and possess chemical structures including the hormone estradiol, suggesting that their biological functions may be identical [60]. Soy isoflavones can protect against hormone-dependent malignancies including breast and prostate, and reduce the risk of osteoporosis, cardiovascular disease, diabetes, and menopausal symptoms [61]. Isoflavones combat cancer by reducing inflammation, counteracting free radicals, promoting programmed cell death, and disrupting signaling pathways such as PI3K/Akt, ERK 1/2, MAPK, and Wnt/β-catenin [62]. Soy isoflavones suppress breast cancer cell progression independently of estrogen receptors, reducing hazards in both pre and post-menopausal women [63].

#### Clinical study

A randomized double-blind placebo-control trial was implemented to evaluate the activity of soy isoflavones against breast cancer for a duration of two years. Premenopausal women (30–42 years) were randomized to receive isoflavones (136.6 mg aglycone equivalents, n = 99) versus a placebo (n = 98) over a period of five days per week for up to 2 years. Breast composition alters, assessed via magnetic resonance imaging (MRI) at baseline and annually, were analyzed using square root transformation and linear mixed-effects regression models. Soy isoflavones were found to lower federated gradient boosting trees (FGBT), a breast cancer risk biomarker, in premenopausal women who participated in the clinical trial. Furthermore, calcium showed mild effects on body mass index (BMI) and breast fat, indicating that soy consumption may have prospective health advantages in the prevention and management of breast cancer [64].

#### Mechanistic view against breast cancer

**Figure 6:**
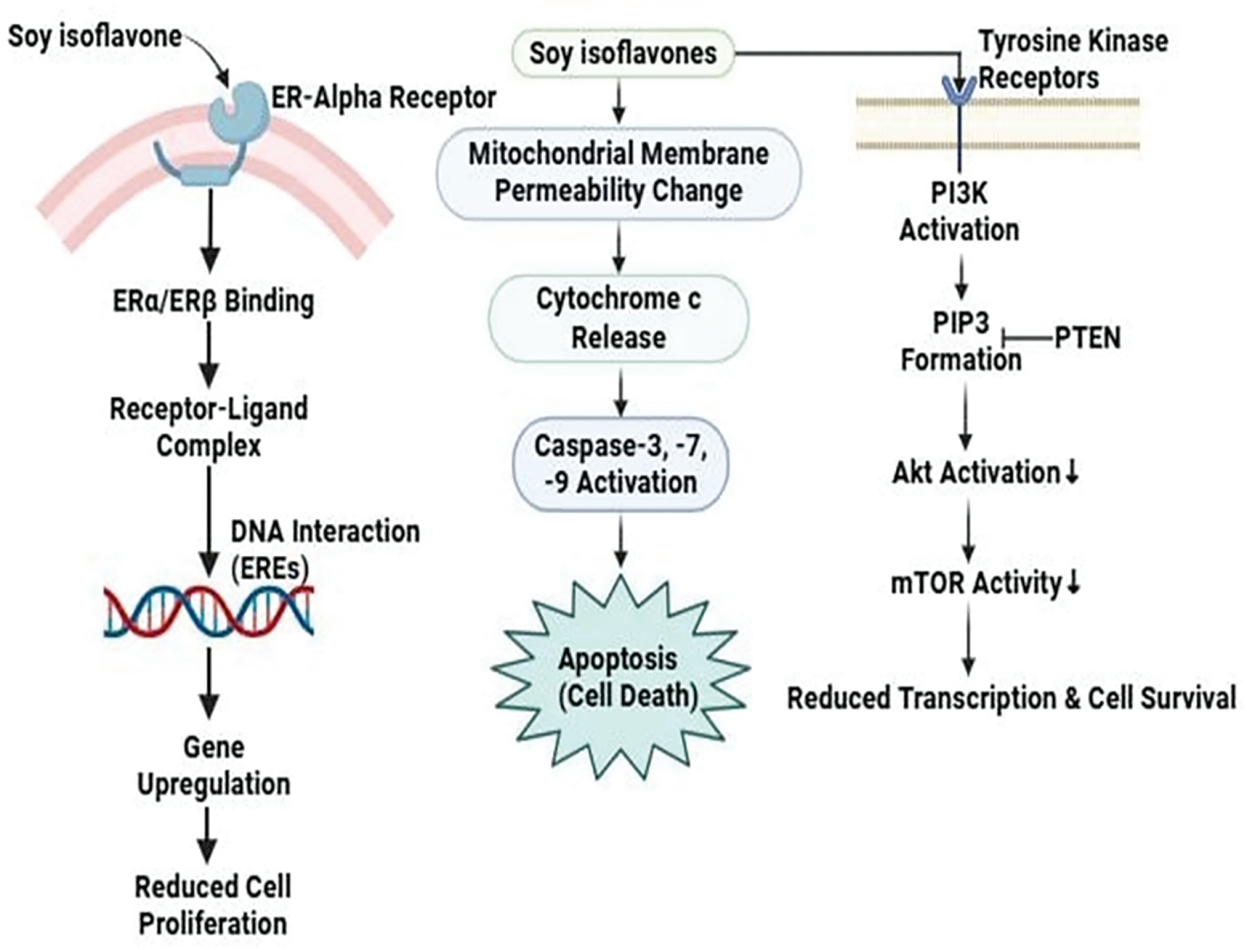
The mode of action of soy isoflavones against breast cancer: Soy isoflavones reduce cell proliferation and promote apoptosis through mitochondrial cytochrome c release, inhibiting breast cancer by directing toward estrogen receptors and the PI3K/Akt/mTOR pathway. To further control the proliferation of cancer cells, they further decrease mTOR activation and alter IGF-1R signaling [PI3K/Akt/mTOR = phosphoinositide 3-kinase (PI3K), protein kinase B (Akt), mammalian target of rapamycin (mTOR). PTEN= Phosphatase and Tensin Homolog, a tumor suppressor. PIP3= phosphatidylinositol (3,4,5)-trisphosphate]. Credit: bioRender.

Soy isoflavones exhibit potent anticancer properties in breast cancer by specifically targeting estrogen receptor (ER) and PI3K/Akt/mTOR signaling pathways. Their effects range from promoting pro-apoptotic effects through complex interactions with mitochondria-dependent signaling pathways in the T47D breast cancer cell line [63]. In a set of differentiated actions, it can effectively inhibit aberrant cell proliferation by targeting transcriptional genes associated with breast cancer cell proliferation and survival. First, isoflavones that target ERα and ERβ form strong receptor-ligand complexes that bind to the ligand-binding domain, triggering interactions with estrogen response elements (EREs) located on DNA and initiating genomic pathways. The upregulation of specific genes resulting from this process contributes significantly to the downregulation of factors that promote breast cancer cell proliferation [65]. Apoptosis is induced by soy isoflavones through the mitochondrial route. Because of them, cytochrome c is excreted into the cytosol, and the mitochondrial membrane permeability is altered. Apoptosis, cell survival, and the modulation of the balance between programmed death and Upstream effector caspase cascades are all regulated by cytochrome c in breast cancer cells [66]. However, isoflavones included in soy stimulate the phosphorylation of the insulin-like growth factor 1 receptor (IGF-1R). Subsequent signaling events along the PI3K/Akt pathway are set off via this event. The Akt cascade is set in motion when Akt is phosphorylated, and PTEN, a lipid phosphatase, inhibits Akt as well as acts as a down regulator by dephosphorylating PIP3 [67]. As a result, protein synthesis is reduced and cell proliferation is regulated, as mTOR activation is suppressed. This well-structured molecular array shows how Soy isoflavones affect cell proliferation and survival via a complicated regulatory process including PI3K/Akt, mTOR, and IGF-1R [68] (Fig. 6).

### Viscum album L.

*Viscum album* L., commonly referred to as European mistletoe, is a popular evergreen plant with hemi parasitic traits that is primarily widespread across Europe, northwest Africa, and southwest and central Asia [69]. The chemical composition of the *Viscum album* differs with host tree species, harvest time, and manufacturing process. Major phytochemicals, such as lectins and vincetoxins, are indispensable in cancer management due to their apoptotic and cytotoxic activities [70]. The plant’s extracts mainly aqueous ones are commonly used in both folk and conventional medicine. They are utilized for various reasons, including the management of conditions such as hypertension and arthritis [71]. They are also well known for their anti-diabetic [72] and hepatoprotective actions [73]. Applications of *V. album* might be appropriate for diminishing the burden of symptoms in breast cancer patients undergoing anticancer therapy [70].

#### Clinical study

A cohort study was conducted in Switzerland and Germany where 1442 breast cancer patients were incorporated; 710 were part of the test population and 732 were in the control population. For a minimum of three months, the test group underwent subcutaneous injections of *V. album* extract alongside standard therapy, whereas the control population was administered standard therapy alone. In the *V. album* extract group, various indications disappeared more often, and the overall survival predictions were considerably extended (adjusted mortality hazard ratio (95% CI): HR = 0.46 (0.22-0.96), *p* = 0.038). The outcomes of the study confirm that using a standardized extract of *V. album* as an adjuvant treatment for patients with primary non-metastatic breast cancer is safe. According to the findings, there has been a noticeable drop in adverse drug reactions (ADRs) associated with concomitant conventional therapy. In addition, relative to population control, the *V. album* extract group displayed signs of a longer overall survival [74].

#### Mechanistic view against breast cancer

**Figure 7:**
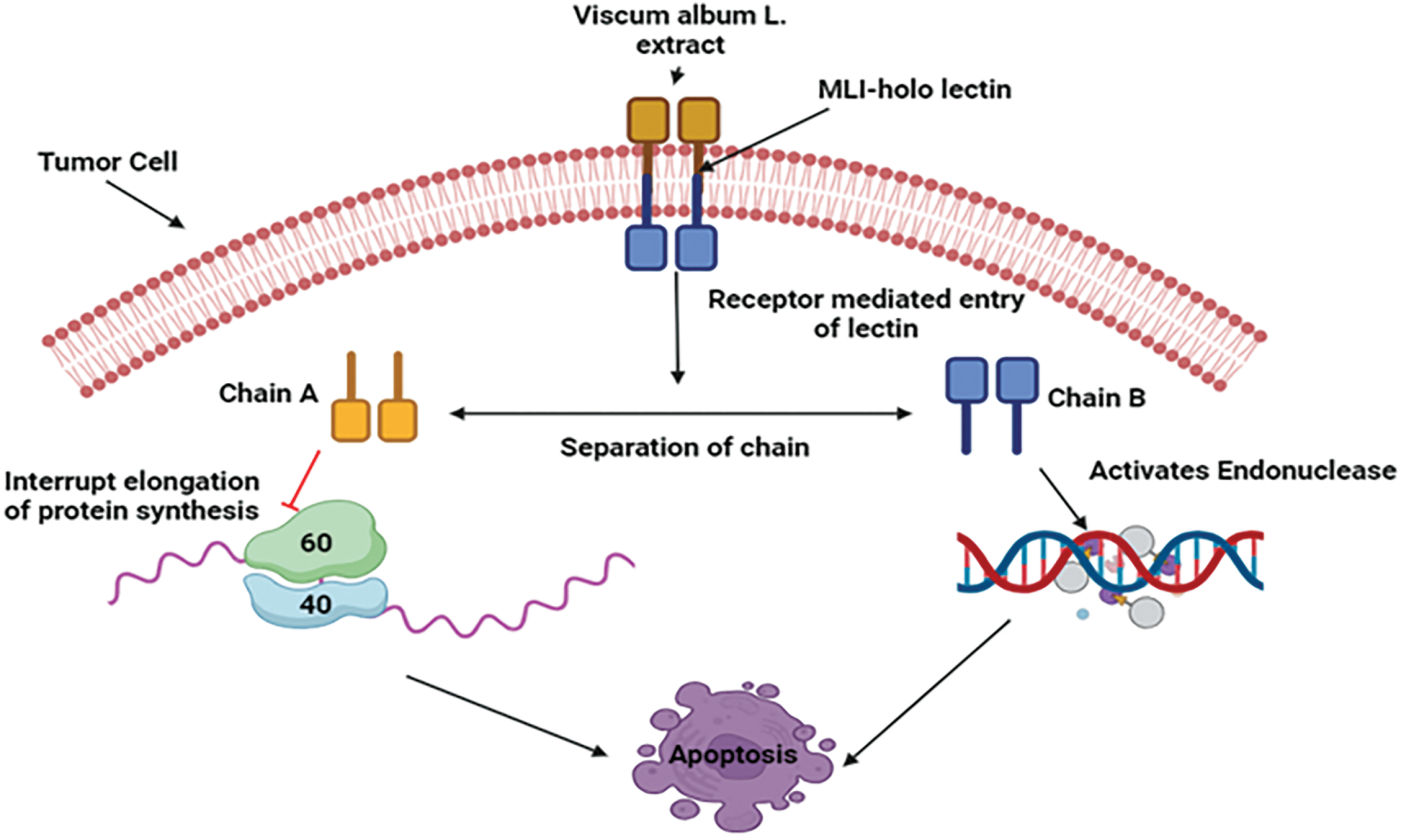
Mechanistic view of *V. album* against breast cancer: By triggering apoptosis and the release of immune cytokines, *V. album* has anticancer properties. By attaching to receptors, mistletoe lectin I (ML-I) specifically destroys cancer cells by stopping protein synthesis in the A-chain and activating endonuclease in the B-chain, which causes cell death (MLI—Mistletoe Lectin I, 60S—60S Ribosomal Subunit, 40S—40S Ribosomal Subunit). Credit: bioRender.

Apoptosis and cytokines produced from immune cells are the main processes behind the anti-cancer effect of *V. album* based on preclinical studies. Through controlling gene expression, the plant causes tumor cells to undergo apoptosis by activating caspases and ultimately causing cell death. Mistletoe lectin I, also known as *viscum album* agglutinin agglutinin-I (VAA-I) is the paramount galactoside-specific lectin. When lectins interact with their potential receptors, their unique sugar-binding specificities are essential in explaining why lectins are selectively lethal to cancer cells. This further emphasizes the role that A- and B-Chains play in lectins’ cytotoxic effects in breast cancer MDA-MB-361 cell lines [75]. Chain B activates endonuclease, whereas Chain A primarily hydrolyzes the glycosidic bond in the 28S RNA of the 60S ribosome, preventing the protein synthesis’s elongation phase. The cancer cell eventually undergoes apoptosis as a result of this process [76] (Fig. 7).

### Salvia miltiorrhiza

For millennia, people in China and other Asian nations have utilized the roots of the traditional Chinese herb *Salvia miltiorrhiza* (Danshen) to treat cardiovascular disease [77]. Across numerous cancer cells, comprising lines, those from the prostate, lung, breast, leukemia, glioma, and liver, *Salvia miltiorrhiza* promotes cell cycle arrest along with apoptosis, according to recent studies, owing to its wide-ranging growth inhibitory and cytotoxic properties [78–80]. Another study involving *Salvia miltiorrhiza* shows preventive effects on patients with breast cancer. This effect may be related to dihydroisotanshinone I (DT), a refined chemical found in *Salvia miltiorrhiza*, that prompts breast cancer cells to undergo apoptosis and ferroptosis [81].

#### Clinical study

A cohort study that included 79,335 individuals suffering from metastatic breast cancer was conducted in Taiwan from 2000 to 2010. The following categories of individuals were identified based on their usage of *Salvia miltiorrhiza* (danshen): non-users; users based on dosage (84 g or less after diagnosis); and users based on duration (28 days or fewer after diagnosis). The significantly lower mortality rates (*p* < 0.001) among patients who took >84 g of danshen after diagnosis suggest a strong correlation between *Salvia miltiorrhiza* use along improved survival rates among breast cancer patients [81].

#### Mechanistic view of Salvia miltiorrhiza against breast cancer

**Figure 8:**
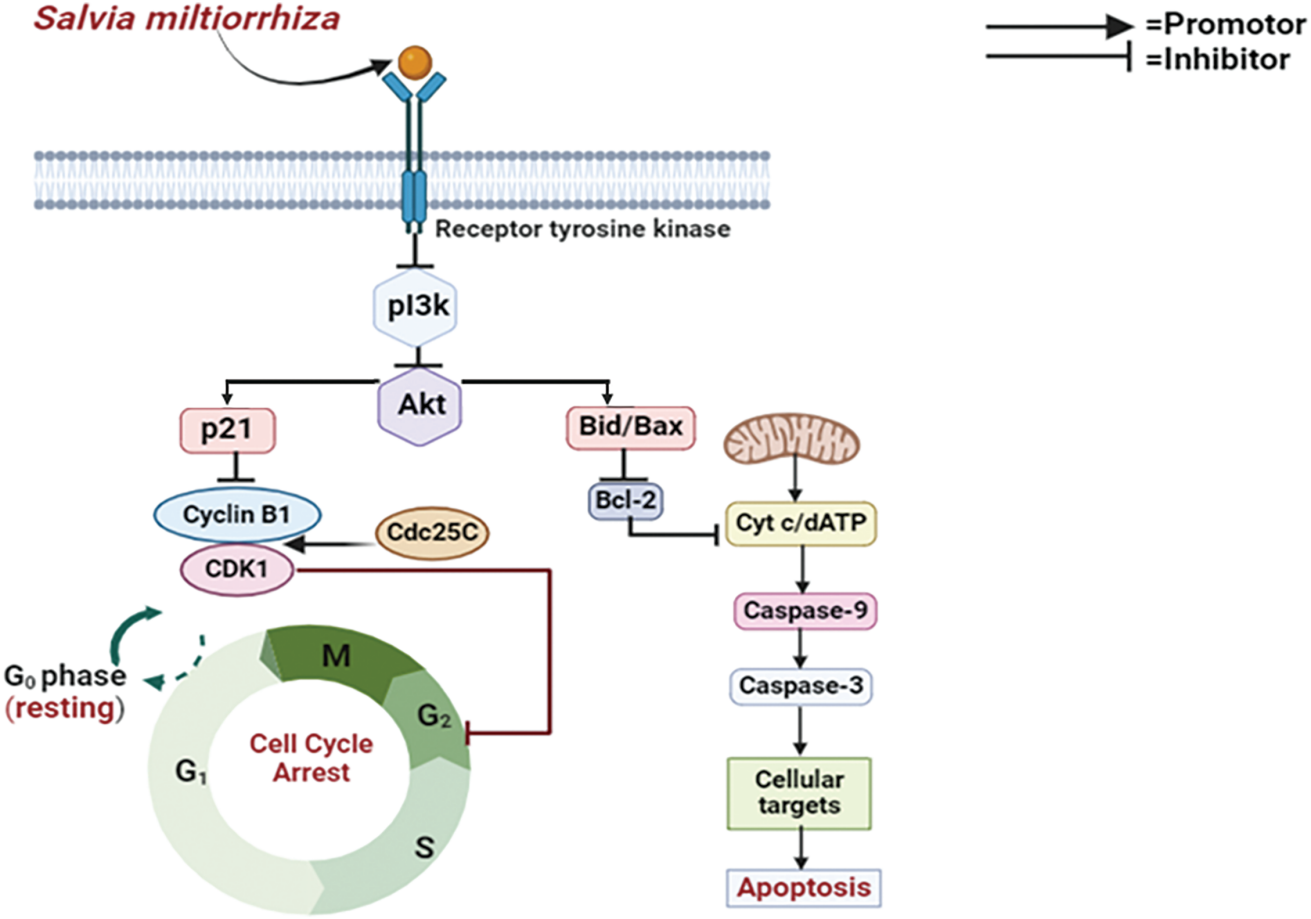
Mode of action of *Salvia miltiorrhiza* (Danshen) against breast cancer: *Salvia miltiorrhiza* inhibits cell growth and survival by targeting the PI3K/Akt pathway, which is consistently dysregulated in malignancies such as breast cancer. It interrupts the signaling cascade by blocking PI3K or Akt, which causes apoptosis and stops the growth of tumors. Additionally, this inhibition causes G1/M or G1 phase arrest by stimulating cell cycle suppressors, particularly p21 and p27 (PI3K—Phosphoinositide 3-Kinase, Akt—Protein Kinase B, p21—Cyclin-Dependent Kinase Inhibitor, CDK1—Cyclin-Dependent Kinase 1, Cdc25C—Cell Division Cycle 25C, Bid/Bax—BH3 Interacting-Domain Death Agonist /Bcl-2-Associated X Protein, Bcl-2—B-Cell Lymphoma 2, Cyt c—Cytochrome c, dATP—Deoxyadenosine Triphosphate, Caspase-9—Cysteine-aspartic Protease 9,Caspase-3—Cysteine-aspartic Protease 3). Credit: bioRender.

Several cancers, including breast cancer, are commonly linked to the deregulation of the PI3K/Akt pathway, which is essential for controlling cell growth, survival, and multiplication. To prevent cancer cells from surviving and proliferating, *Salvia miltiorrhiza* targets important elements of this signaling cascade [68,82]. These inhibitors function about breast cancer by preventing the action of PI3K, a kinase that produces signaling molecules that trigger Akt. Akt, which is the term for protein kinase B in MCF-7 and MCF-7 HER2 cells, is critical for encouraging cell growth and viability. These pathway suppressors stop cancer cells from getting signals that lead to unchecked growth by blocking PI3K or Akt, which interrupts the signaling cascade. Inducing apoptosis, preventing cell cycle proliferation, and eventually slowing tumor growth are the objectives of this focused intervention [83]. Yet again, suppressing PI3K causes the signaling cascade to be disrupted, which has multiple consequences that lead to cell cycle arrest. First off, it might inhibit the synthesis of cyclins, which would hinder the cell cycle phases from progressing as intended. Moreover, cell cycle suppressors like p21 and p27, which stop the cell cycle by blocking cyclin-dependent kinases (CDKs), can be upregulated by PI3K inhibition. Together, these molecular alterations cause either G1/M phase arrest or G1 phase arrest [84] (Fig. 8).

### Curcumin

Curcumin, the primary constituent of turmeric, is well known for its uses in complementary medicine. Curcumin demonstrates a remarkably wide range of advantageous characteristics, involving anti-inflammatory, antioxidant, chemo-preventive, and chemotherapeutic effects [85]. It has drawn a lot of attention as a possible therapeutic compound for both the prophylaxis and management of numerous diseases particularly multiple myeloma, pancreatic cancer, myelodysplastic syndromes, colon cancer, psoriasis, and Alzheimer’s disease [86]. It holds an essential part in breast cancer and supplies a comprehensive approach for halting the growth of breast cancer cells due to its anticancer impact within intricate molecular signaling networks including proliferation, ER, and HER2 pathway, which are the precursors of breast cancer [87].

#### Clinical study

A study including 150 women diagnosed with progressed-stage and metastatic breast cancer was implemented to ascertain the safety and potency of curcumin in the management of breast cancer. The women received intravenous treatment for 12 weeks, with a 3-month follow-up, either paclitaxel (80 mg/m^2^) combined with placebo or paclitaxel combined with curcumin (CUC-1^®^, 300 mg solution, once per week). During the 4 weeks of observation, the objective response rate (ORR) for curcumin was substantially greater than that of the placebo (51% vs. 33%, *p* < 0.01), according to the intention-to-treat (ITT) analysis. Three months after the treatment ended, curcumin was found to have a better effect than a placebo provided to each of the patients who had undergone the treatment regime and all individuals participated in the analysis [88].

#### Mechanistic view of curcumin against breast cancer

**Figure 9:**
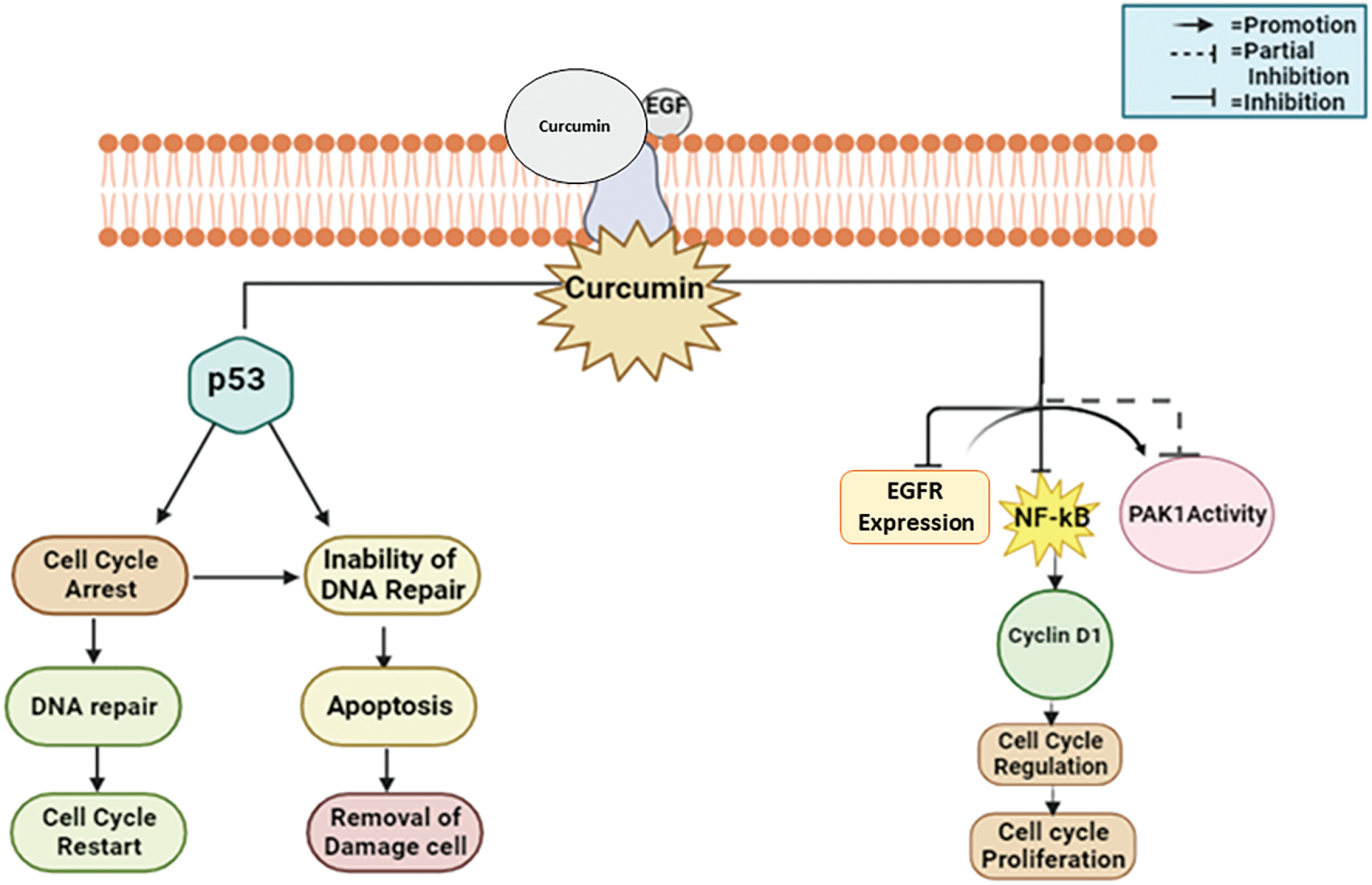
The mode of action of curcumin against breast cancer: Curcumin suppresses tumor expansion and angiogenesis by initiating apoptosis, cell cycle arrest, and downregulating CDK inhibitors. It also modifies signaling proteins including Akt and mTOR [EGF—Epidermal Growth Factor, EGFR—Epidermal Growth Factor Receptor, NF-κB—Nuclear Factor Kappa B, PAK1—p21-Activated Kinase 1, p53—Tumor Suppressor Protein 53]. Credit: bioRender.

Curcumin largely inhibits pro-cancer processes for example inflammation, angiogenesis, and metastasis, and activates apoptotic pathways in cancer cells to produce anticancer effects [89]. It frequently appears in breast cancer tissues that the tumor suppressant protein p53 is under-expressed along with CDKs overexpressed [90]. Many cell cycle control proteins, for example, the CDK inhibitors p21, p27, and p57, are downregulated concurrently [91]. Thus, curcumin inhibits the growth of breast cancer cells by a variety of mechanisms, particularly triggering cell cycle arrest and p53-dependent apoptosis; altering the expression of signaling proteins such as Akt, PI3K, Ras, and mTOR; downregulating transcription factors; and suppressing tumor expansion and angiogenesis in MCF-7 breast cancer cell lines [92] (Fig. 9).

### Green tea

Green tea, from *Camellia sinensis* leaves, is an ancient beverage [93]. The countless positive health impact of green tea is the reason for its prominence in nutraceuticals and functional foods. Green tea extract contains potent antioxidants, notably catechins like epigallocatechin-3-gallate, effective against oxidative stress [94]. Green tea provides various health benefits, including cancer and cardiovascular disease prevention [95], anti-inflammatory [96], antiarthritic [97], antibacterial [98], and antiviral activities [99], as well as antioxidant [96] and neuroprotective advantages [100]. It is also recognized for its cholesterol-lowering and antiangiogenic properties. High consumption of green tea has been connected to a decreased risk of cancer, according to a previous study [101]. Green tea includes chemicals called catechins, for example, epicatechin gallate, epicatechin gallate, and epicatechin-3-gallate, which have been shown to have little adverse effects while potentially preventing the formation of tumors [102,103]. The extract or green tea has been proven effective in treating multiple variety of cancers involving breast, colorectal, prostate, ovarian, liver, lung, lymphoma, gastric, and pancreatic cancer in clinical trials [104–108]. Green tea polyphenols, significantly epigallocatechin gallate (EGCG), have been discovered to impede the expansion, reproduction, movement, and formation of blood vessels in breast cancer cells, demonstrating the prominent for both hindering and managing the disease [109].

#### Clinical study

As a part of the Phase IB trial evaluating the effectiveness of Polyphenol E, conducted with 40 women who had endured adjuvant treatment for stages I–III breast cancer, its effectiveness was evaluated. Participants received Polyphenon E (400 mg, n = 16; 600 mg, n = 11; or 800 mg, n = 3) or placebo (n = 10). Notable reductions in serum hepatocyte growth factor (HGF) levels at 2 months, along with decreases in VEGF levels at 2 and 4 months, were observed in the Polyphenon E group, alongside a trend towards decreased serum cholesterol. These findings imply that green tea polyphenols might contribute to suppressing growth factor signaling, angiogenesis, and lipid metabolism in breast cancer therapy [110].

#### Mechanistic view against breast cancer

**Figure 10:**
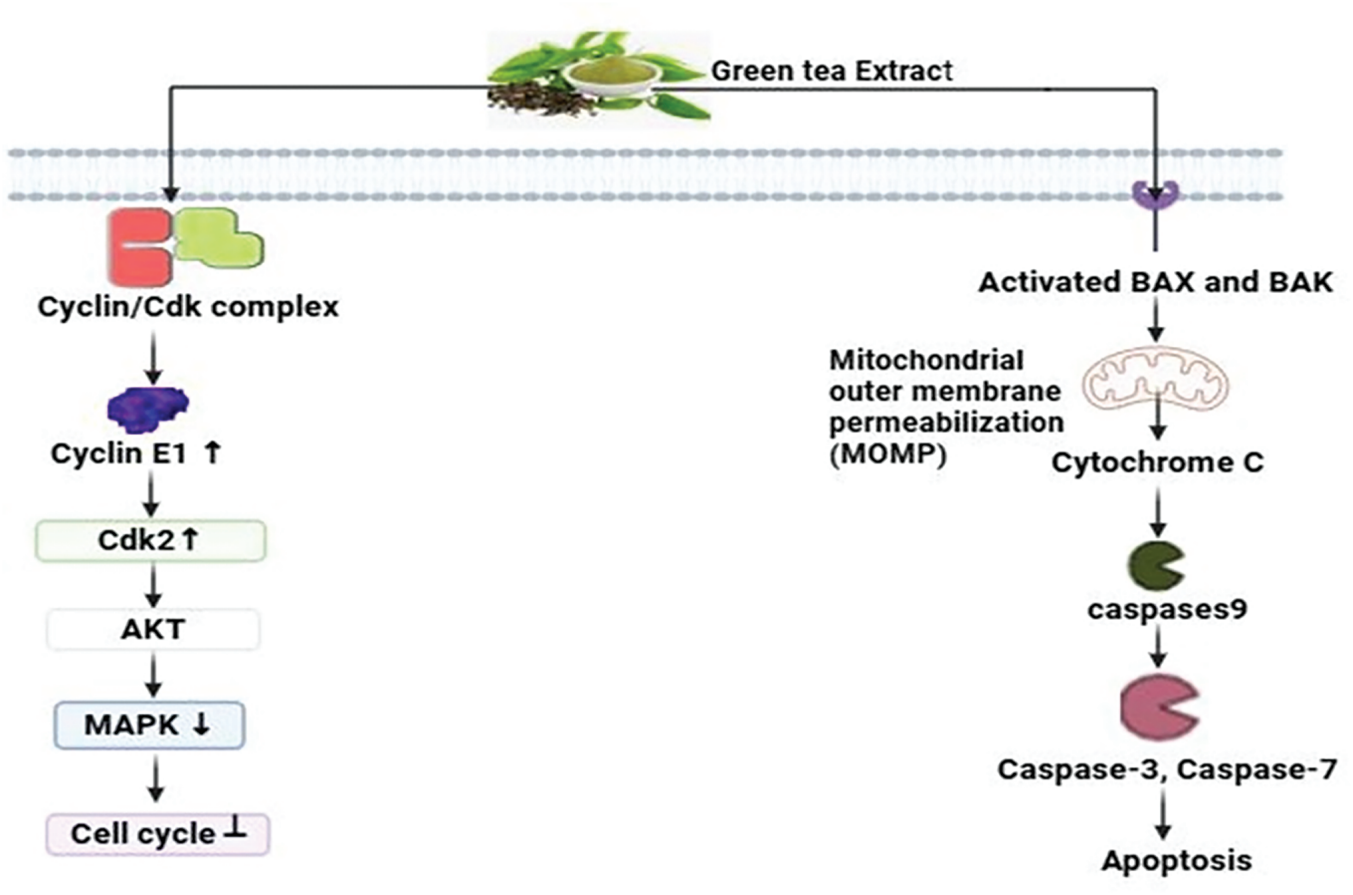
The mode of action of green tea extract against breast cancer: By inhibiting the MAPK and AKT pathways and inducing cell cycle arrest at the G1/M and G2/M checkpoints, green tea polyphenols (GTPs), particularly EGCG, prevent breast cancer. They demonstrate their potential in the prophylaxis and management of breast cancer by activating the pro-apoptotic proteins Bak and Bax, which results in caspase activation and mitochondrial permeabilization (AKT= Protein Kinase B, MAPK = Mitogen-Activated Protein Kinase, Epigallocatechin gallate = EGCG, CDK= Cyclin-dependent kinase, ↑ = increase, ↓ = decrease). Credit: bioRender.

Green tea polyphenols (GTPs), particularly EGCG, have various impacts on cancer cells in breast cancer studies. They intricately control cell cycle advancement by acting on essential proteins like cyclin E1, cyclins, and cyclin-dependent kinase increases, causing cell cycle halt at critical checkpoints including G1/M and G2/M transitions in MCF-7 human breast cancer cells [111]. Moreover, GTPs intimately decrease essential signaling pathways involving MAPK and AKT, hence inhibiting cancer cell growth [112]. Furthermore, Green tea polyphenols initially activate the pro-apoptotic proteins Bax and Bak subsequently triggering the pathways involving mitochondrial permeabilization, triggering a series of cellular processes such as chromatin condensation, release of cytochrome c, and subsequent activation of caspase-3, -7, and -9 [113]. The coordinated mechanisms highlight the potential of GTPs, especially EGCG, as viable options for breast cancer prophylaxis and management (Fig. 10).

### Scutellaria barbata (BZL101)

*Scutellaria barbata* or Barbat skullcap is a *Lamiaceae* family perennial herb native to the damp flatlands of southeastern China which contains over 350 species. *Scutellaria* occupies one of the most giant genera within the *Lamiaceae* family. In late summer, the plant reaches its peak bloom, and drying its above-ground portions enhances its therapeutic benefits [114,115]. It has been proven via research that Scutellaria and its active principles display a variety of pharmacological actions. These actions include antioxidant, antimicrobial, antifeedant, phytotoxic, acaricidal toxicity, antibacterial, anti-inflammatory, and analgesic properties [116]. *Scutellaria barbata* exhibits notable anti-tumor effects in numerous types of cancers, including skin, lung, uterine leiomyoma, colorectal, breast, and cervical cancers [117]. *Scutellaria barbata* (BZL101), which is being intensively researched for the treatment of breast cancer, consistently exhibits cytotoxic effects on ER+ and ER−breast cancer types and inhibits over 50% in several cell lines [118].

#### Clinical study

A Phase I clinical trial was implemented to scrutinize the effectiveness of BZL101, a derivative derived from *Scutellaria barbata*, in treating advanced breast cancer. The extract has promising efficiency by inhibiting the advancement of cancer cells. Within the study including 21 patients, BZL101 demonstrated a notable degree of tolerance, since there were no instances of severe side effects (grade III or IV) recorded. Further studies are required to ascertain the potency of BZL101 as a possible therapy for invasive breast cancer [119].

#### Mechanistic view against breast cancer

**Figure 11:**
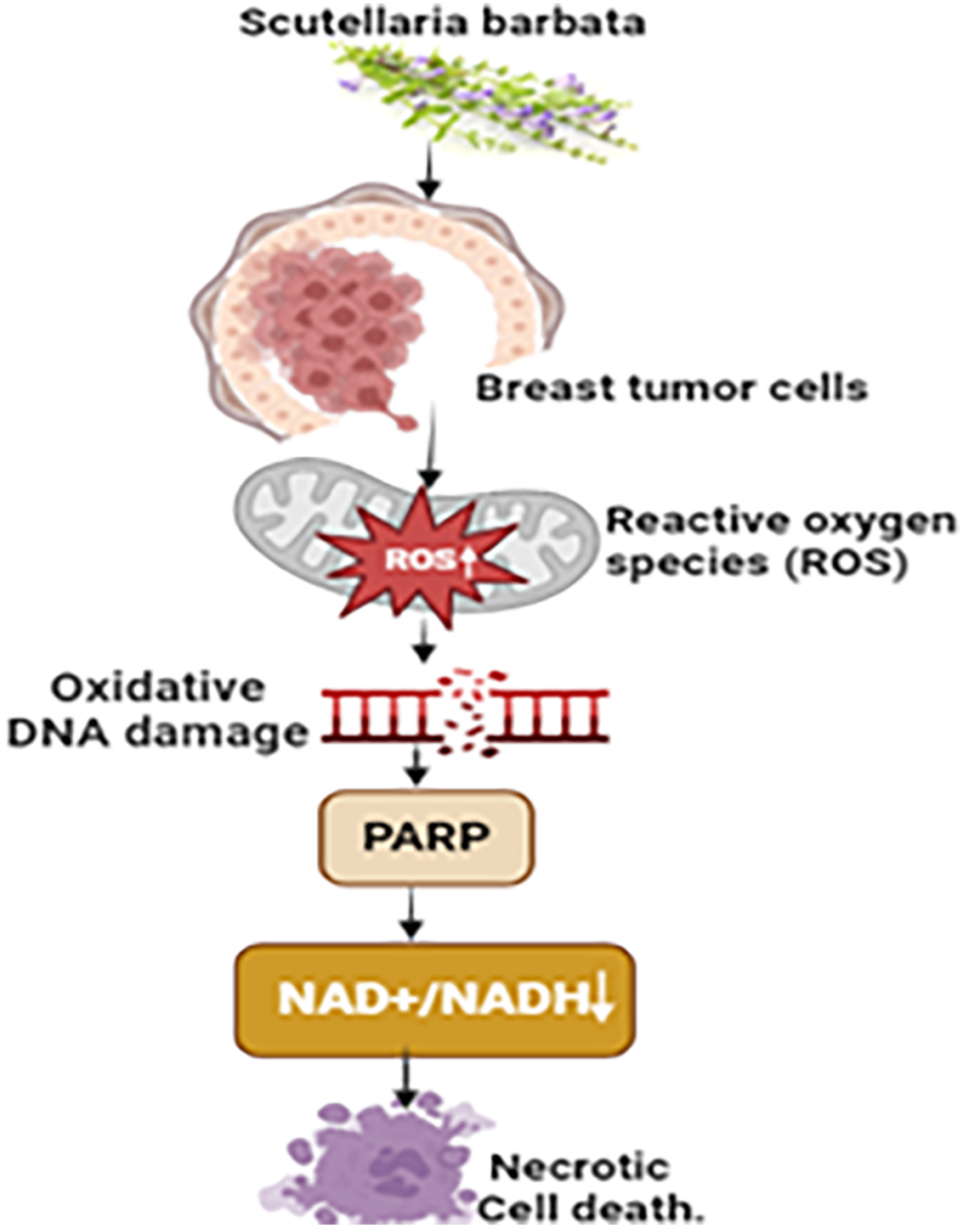
The mechanism of action of *Scutellaria barbata* against breast cancer: Through oxidative DNA damage and excessive PARP activation, the BZL101 extract from *Scutellaria barbata* exhibits specific cytotoxicity against breast cancer cells by dramatically raising reactive oxygen species (ROS) levels. As a result, there is a decrease in NAD+/NADH levels, which are essential for aerobic glycolysis in tumor cells. This contributes to an energy collapse and ATP depletion, which finally results in necrotic cell death (NAD+/NADH= (Nicotinamide Adenine Dinucleotide) & (Nicotinamide Adenine Dinucleotide, Reduced), PARP=Poly (ADP-ribose) Polymerase, “↑” = Increase and “↓” = Decrease). Credit: bioRender.

The BZL101 extract derived from *Scutellaria barbata* exhibits notable cytotoxic properties against breast cancer cells. It displays a remarkable selectivity by specifically enhancing the amounts of reactive oxygen species (ROS) within tumor cells while having a comparatively milder effect on normal cells. BZL101 induces a significant increase in endogenous ROS within the MCF-7 human breast tumor cells, resulting in considerable oxidative DNA impairment and the overactivation of poly (ADP-ribose) polymerase (PARP) [120]. Consequently, this series of events results in a substantial reduction in NAD+/NADH levels, which is especially crucial for tumor cells that rely on aerobic glycolysis. The fast decrease in NAD+/NADH levels, mediated by PARP, triggers a reaction in tumor cells, leading to the depletion of ATP reserves and finally resulting in an energy collapse [121]. This mechanism results in the death of necrotic cells, which highlights the particular and focused cytotoxicity of BZL101 against breast cancer cells (Fig. 11).

### Trametes versicolor

Turkey tail, also known as *Trametes versicolor*, is a widespread Basidiomycota fungus that may be found on deciduous and coniferous trees, such as oak and pine, and it displays basidium on the trunks of these trees throughout the entire year. *Trametes versicolor*, a renowned medicinal mushroom, is recognized globally [122]. Antioxidant-rich *Trametes versicolor* mushrooms strengthen the body’s defenses against free radicals along with ROS, helping to prevent illnesses including cancer, diabetes, cardiovascular problems, and neurological disorders. It decreases the expression of anti-apoptotic genes as well as enhances the expression of pro-apoptotic genes [122]. *Trametes versicolor* extracts have remarkable efficacy in regulating immune responses to suppress the advancement of breast cancer cells and other forms of cancer [123].

#### Clinical study

A phase I trial was implemented on 11 breast cancer patients to evaluate the effectiveness of *Trametes versicolor* (Tv) at doses of 3, 6, and 9 g after radiation treatment. The results indicated that Tv demonstrated both safety and the potential to efficiently restore the immune system. There is a positive correlation between higher doses and faster recovery, indicating that Tv is a reliable form of immunotherapy. Phase II studies are necessary to investigate its potential to enhance immunity and reduce the recurrence of breast cancer [123].

#### Mechanistic view against breast cancer

**Figure 12:**
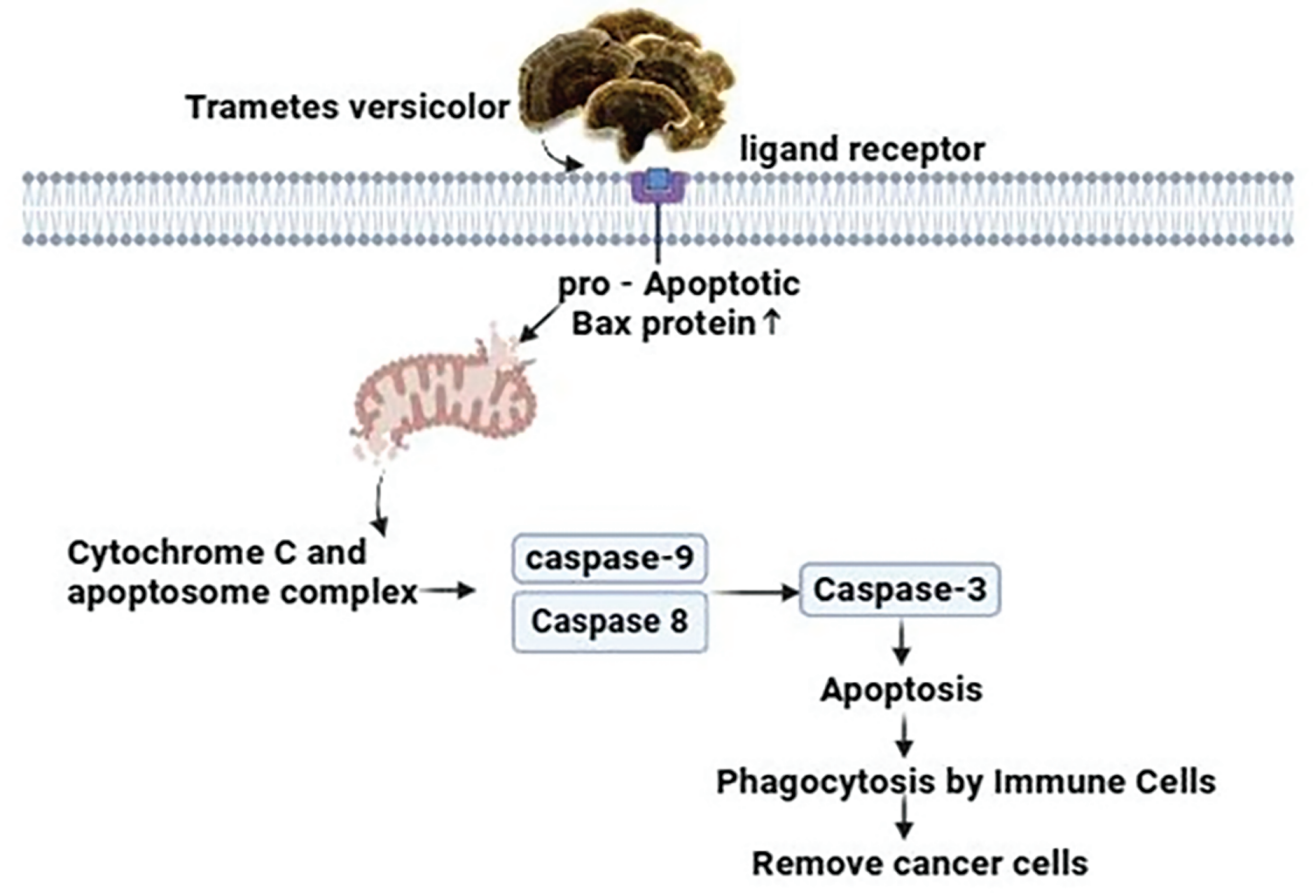
The mechanism of action of *Trametes versicolor* against breast cancer: By boosting pro-apoptotic Bax protein expression, activating cell receptors, and improving mitochondrial membrane permeability, extracts from *Trametes versicolor* inhibit breast cancer cells. Apoptosis, caspase activation, and the excretion of cytochrome c are the results of this. Furthermore, the apoptotic cancer cells are phagocytosed and destroyed by immune cells that have been activated. Credit: bioRender.

*Trametes versicolor* extracts employ an intricate approach to counteract breast cancer cells, requiring advanced cellular interactions and molecular signaling cascades [124]. The extracts stimulate the receptors that allow access into the cell, resulting in an enhancement in the expression of the pro-apoptotic Bax protein in the T47D breast cancer cell line. This protein, consequently, promotes the permeability of the mitochondrial membrane. This synchronized activity initiates the liberation of cytochrome c, which then combines with apoptosome to stimulate programmed cell death [125]. This process activates caspases and finally results in apoptosis. Subsequently, the immune cells are stimulated to initiate phagocytosis, which involves engulfing and eliminating apoptotic cancer cells [66] (Fig. 12).

### Limonene

Limonene, chemically known as 1-methyl-4-(1-methyl phenyl)-cyclohexene, is a cyclic monoterpene widely found in the essential oils in several plants, especially in citrus fruits for example oranges, lemons, and grapefruits [126]. It demonstrates numerous pharmacological actions, including antioxidant, antimicrobial, and anti-inflammatory effects [127]. Furthermore, limonene has drawn attention to its possible anticancer effects due to its capability to suppress tumor expansion and induction of apoptosis [128]. Regarding breast cancer, limonene exhibits promising anti-breast cancer activities, presenting itself as a paramount therapeutic agent in the treatment of breast cancer [128]. It has been demonstrated to modulate the duration of the cell cycle, resulting in cell cycle arrest and eventually apoptosis in breast cancer cells [129].

#### Clinical study

A single-arm, open-label intervention test was carried out at the University of Arizona Cancer Center, (Tucson, AZ). 43 individuals diagnosed with early-phase breast cancer and planned for surgical procedures were included in this limonene intervention trial. Patients were given *2 g of limonene daily* for a period of two to six weeks before surgery. For the determination of the limonene-initiated alterations in systemic and tissue biomarkers of the breast malignancy, blood, and breast tissue samples were analyzed. The result of the intervention of limonene was a 22% decrement in the expression of cyclin D1 (*p* = 0.002) whereas the tissue Ki67 and cleaved caspase-3 showed negligible alteration. Due to the significant reduction of the cyclin D1, it can be concluded that limonene may lead to the arrest of the cell cycle and suppress the expansion of cells in breast tumors [130].

#### Mechanism of limonene against breast cancer

**Figure 13:**
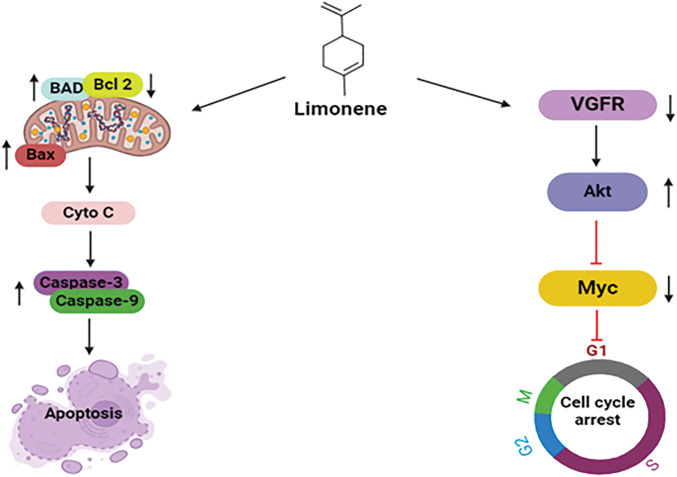
Mechanistic view of limonene against breast: Through the upregulation of BAD, inhibition of anti-apoptotic Bcl-2, and release of pro-apoptotic Bax, which results in cytochrome c release and caspase activation, limonene promotes programmed cell death in breast cancer. Additionally, it halts the growth of cancer cells by downregulating VEGF and blocking Akt and Myc, which results in G1-phase cell cycle arrest (BAD = Bcl-2-associated Agonist of Cell Death, Bcl2 = B-cell Lymphoma 2, BAX = Bcl-2-associated X protein, Cyto C = Cytochrome C, VGFR = Vascular Endothelial Growth Factor Receptor, Akt = Protein Kinase B, Myc = Myelocytomatosis Oncogene). Credit: bioRender.

Limonene orchestrates apoptosis, a mechanism of programmed cell death, by controlling key apoptotic proteins which are BAD, Bcl-2, and caspases, along with cytochrome c. Limonene’s action on BAD is achieved by upregulating its activity, resulting in the suppression of anti-apoptotic Bcl-2 proteins by forming a complex with it [128]. Pro-apoptotic proteins, namely Bax are freed up by this inhibition and can permeabilize the mitochondrial membrane. In consequence, cytochrome c is discharged into the cytoplasm of the mitochondria, where it activates the caspase enzymes. Limonene further enhances this cascade by promoting the initiation of caspases, particularly caspase-3 and caspase-9, which eventually execute the apoptotic process through the fragmentation of essential cellular components [131]. Limonene initiates G1-phase cell cycle arrest by targeting vital signaling molecules in the BT483 human breast cancer cell line. It downregulates vascular endothelial growth factor (VEGF), thereby attenuating the activation of Akt. Additionally, limonene inhibits downstream cell cycle regulatory pathways by suppressing the expression of Myc. Limonene suppresses cancer cell multiplication via the modulation of VEGF, Akt, and Myc, which may have healing effects in treating breast cancer. These multifaceted mechanisms highlight limonene’s capability as a promising therapeutic candidate t against breast cancer [132] (Fig. 13).

### Flaxseed

*Linum usitatissimum*, also known as flaxseed or linseed, grows mainly in 50 countries in the Northern Hemisphere and has been used for food and fiber [133]. It is gaining eminence as a mainstay supplier of phytochemicals within the field of functional foods. Apart from its well-known richness in α-linolenic acid oil as well as lignans, flaxseed constitutes a crucial reservoir of top-tier protein and soluble fiber, promising significant promise as a prolific origin of phenolic compounds [133,134] The possible health benefits of flaxseed oil, fibers, and lignans include decreasing the risk of osteoporosis, arthritis, cancer, diabetes, atherosclerosis, cardiovascular disease, and neurological and immune-mediated diseases [135]. Flaxseed halts the expansion and metastasis of breast cancer due to its lignans and oil components [136].

#### Clinical study

A randomized double-blind placebo-controlled study was implemented to ensure the impact of dietary flaxseed on postmenopausal breast cancer individuals. Patients were allotted casually to consume either a 25 g flaxseed-containing muffin (n = 19) or a control placebo muffin (n = 13) for a period of 32 & 39 days respectively. At the time of diagnosis, the tissue of the tumor was investigated for the rate of tumor cell growth (ki-67 labeling index, primary endpoint), c-erB2 expression, apoptosis, and estrogen & progesterone receptor levels. Additionally, urine excretion of lignans was scrutinized for 24 h. In the flaxseed group, notable reductions were observed in the Ki-67 labeling index (34.2%; *p* = 0.001) and c-erbB2 expression (71.0%; *p* = 0.003), along with a significant increase in apoptosis (30.7%; *p* = 0.007), whereas no such deviations were monitored in the placebo group. Additionally, a potential increase in lignan excretion in urine in the flaxseed group. So consuming dietary flaxseed holds promise for reducing tumor growth among breast cancer patients [137].

#### Mechanistic view against breast cancer

**Figure 14:**
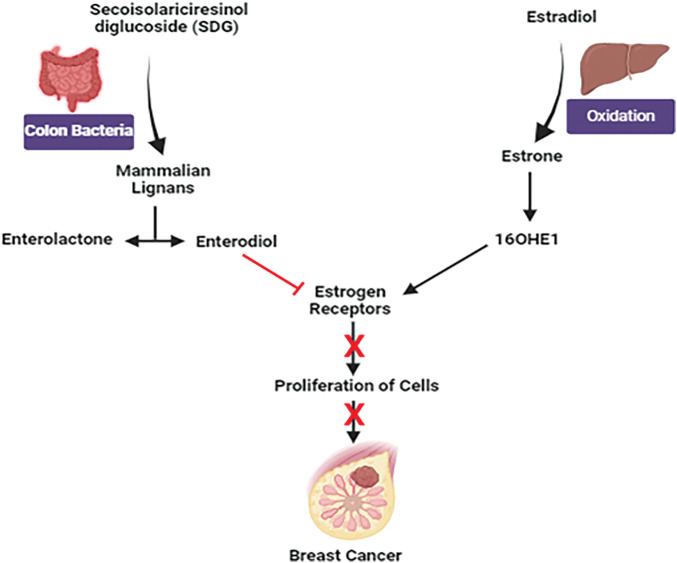
Mechanistic view of flaxseed against breast cancer: Estradiol undergoes oxidation to produce estrone and elevated levels of 16α-hydroxy estrone (16OHE1) are associated with an enhanced susceptibility to breast cancer. Enterolactone and enterodiol, which bind to estrogen receptors and block estrogen action, are produced from secoisolariciresinol diglucoside (SDG) from flaxseed and may slow the expansion of ER+ breast cancer cells. Credit: bioRender.

Estradiol, the biologically active form of estrogen in our body, oxidizes predominantly into estrone in the liver. Further, estrone can be altered into two metabolites where 16α-hydroxyestrone (16OHE1) is mainly responsible for uncontrolled cell proliferation in the breast [138]. Women producing more 16OHE1 are more vulnerable to breast cancer. Secoisolariciresinol diglucoside (SDG), the predominant lignan in flaxseed, contributes around 95% of seed lignan content [139]. After ingestion of SDG lignan, it is altered into mammalian lignans, enterolactone, and enterodiol by colon bacteria. Having structurally similar to estrogen, they have weak estrogenic activity [140]. Due to structural similarity, they bind to estrogen receptors, preventing estrogen binding to the receptors and hence inhibiting the growth in T47D: A18 cell line. Breast tumors containing estrogen receptors (ER+) are more likely to respond to hormonal treatment [141] (Fig. 14).

## Future Perspective and Concluding Remarks

Breast cancer is a complex and widespread cancer that has become one of the leading prevalent and alarming health problems around the world. Owing to the intricacy of breast cancer, new, targeted treatment alternatives must be regularly uncovered, emphasizing the significance of advancements in both research and methods of therapy. Although conventional breast cancer treatments have proven effective, they come with significant drawbacks that have sparked a growing interest in natural products as potential transformative elements in the industry. Moreover, clinically validated natural products can revolutionize breast cancer management and usher in a new era of customized personalized medicine. The ability of natural products, which are rich in bioactive chemicals, to customize treatments to the distinct genetic and molecular characteristics of individual breast tumors is being studied. This tailored strategy can maximize the benefits of treatment while reducing side effects, opening the door to a more complex and efficient paradigm in treating breast cancer. Concurrently, the blend of immunotherapy and natural products appears to be a ray of hope, as it employs the immunomodulatory qualities of substances derived from marine and medicinal plants to strengthen the body’s innate protection against breast cancer cells. With the increasing investigation of modern drug delivery methods, which includes the use of nanotechnology, the potential of precisely targeting natural substances to breast cancer cells becomes realistic. The purpose of this innovation is to minimize collateral damage to healthy tissues while optimizing therapeutic impact. Furthermore, the evolution of breast cancer treatment embraces combination therapies that integrate natural products with established treatments, offering a multifaceted approach to improve efficacy and mitigate side effects. Yet again, this study shows that biomarkers contribute significantly to the advancement of breast cancer through a variety of mechanisms. Hence, the identification of biomarkers continues to be essential in directing toward the development of personalized treatments suited to each patient’s unique needs and guaranteeing that those who stand to gain the most from natural product therapies receive them. Key challenges to including these natural products in therapeutic regimens include poor bioavailability, variability in composition, unclear mechanisms, and potential drug interactions. Limited clinical trials and regulatory barriers further hinder their integration into breast cancer treatment. With continuous clinical research aimed at verifying the protection and efficiency of natural products, the way to obtain regulatory approval and integrate these treatments into conventional breast cancer care procedures is becoming more evident. With these considerations, the incorporation of natural products in treating breast cancer exhibits its potential as a way to provide patients with tailored, less invasive, and possibly more effective therapeutic options. This implies that as our understanding of natural products and their potential benefits expands, we may be able to forecast better outcomes and an enhancement in the quality of life for individuals enduring breast cancer.

## Data Availability

The datasets generated and/or analyzed during the current study are available from the corresponding author on reasonable request.
